# Gene Architecture Facilitates Intron-Mediated Enhancement of Transcription

**DOI:** 10.3389/fmolb.2021.669004

**Published:** 2021-04-21

**Authors:** Katherine Dwyer, Neha Agarwal, Lori Pile, Athar Ansari

**Affiliations:** Department of Biological Science, Wayne State University, Detroit, MI, United States

**Keywords:** transcription, splicing, intron, exon, gene architecture, gene regulation, gene looping

## Abstract

Introns impact several vital aspects of eukaryotic organisms like proteomic plasticity, genomic stability, stress response and gene expression. A role for introns in the regulation of gene expression at the level of transcription has been known for more than thirty years. The molecular basis underlying the phenomenon, however, is still not entirely clear. An important clue came from studies performed in budding yeast that indicate that the presence of an intron within a gene results in formation of a multi-looped gene architecture. When looping is defective, these interactions are abolished, and there is no enhancement of transcription despite normal splicing. In this review, we highlight several potential mechanisms through which looping interactions may enhance transcription. The promoter-5′ splice site interaction can facilitate initiation of transcription, the terminator-3′ splice site interaction can enable efficient termination of transcription, while the promoter-terminator interaction can enhance promoter directionality and expedite reinitiation of transcription. Like yeast, mammalian genes also exhibit an intragenic interaction of the promoter with the gene body, especially exons. Such promoter-exon interactions may be responsible for splicing-dependent transcriptional regulation. Thus, the splicing-facilitated changes in gene architecture may play a critical role in regulation of transcription in yeast as well as in higher eukaryotes.

## Introduction

**FIGURE 1:**
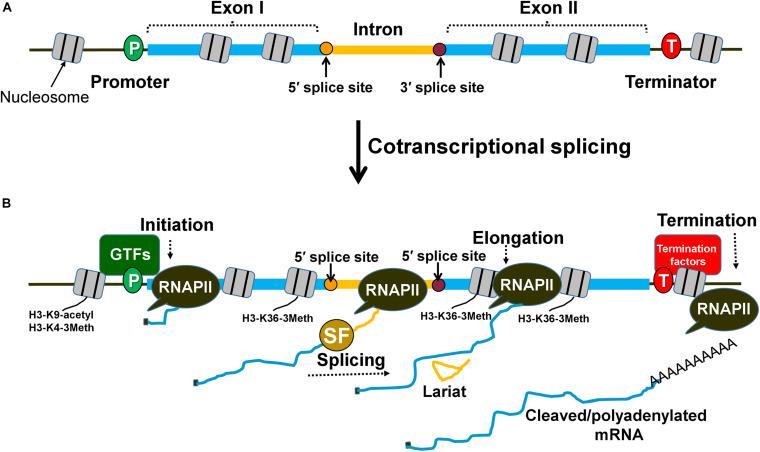
Splicing occurs cotranscriptionally and affects different steps of transcription. **(A)** An intron within the transcribed region is flanked by exon I and exon II. The splice sites are designated as 5′ splice site and 3′ splice site and are the sites for spliceosome assembly during transcription. **(B)** Splicing factors are recruited cotranscriptionally to the intron with the help of the RNAPII carboxy-terminal domain. Spliceosomal assembly on the splice sites can facilitate the stabilization of general transcription factors (GTFs) at the promoter region of the gene and prime nucleosomes with activation marks (H3-K9 acetylation and H3-K4 trimethylation) for initiation. The splicing factors can also interact with transcription elongation factors and influence nucleosome modifications (H3-K36 trimethylation) to promote elongation. Similarly, splicing factors can contribute to enhanced termination of transcription by facilitating the recruitment of termination factors and removal of elongation marks that block effective termination.

Introns are intervening non-coding sequences in eukaryotic genes that are removed from the primary transcripts by the process of splicing (Figure 1). An elaborate splicing machinery is needed to remove introns to form the mature transcript (Padgett et al., 1986). The energy, time consumption, and complex nature of the spliceosome indicate that introns impose a large burden on eukaryotic organisms (Jo and Choi, 2015). However, in spite of all of these drawbacks, introns have been evolutionarily conserved, indicative of their having a fundamental and significant role in the cell (Carmel and Chorev, 2012; Rogozin et al., 2012). In fact, nearly half of all common genetic disorders in humans may be attributed to a disruption in the splicing process (Faustino and Cooper, 2003; Wang and Cooper, 2007; Padgett, 2012). An important question therefore is what is the physiological significance of introns in eukaryotes?

Research conducted during the last few decades has revealed novel biological functions of introns. An apparent advantage conferred by introns is their ability to increase proteomic complexity through the process of alternative splicing, meaning that a single gene can produce multiple isoforms of a protein dependent on cell type and environment (Nilsen and Graveley, 2010). This offers immense proteomic plasticity since the cell can modulate what protein isoform is produced depending on the developmental context, cell type and environmental cues (Marquez et al., 2015; Chaudhary et al., 2019). The presence of introns has also been shown to protect genomic integrity. The process of transcription is accompanied by formation of genotoxic R-loops, which are three stranded nucleic acid structures harboring an RNA-DNA hybrid (Niehrs and Luke, 2020). Intron-containing genes alleviate R-loop formation and in doing so confer genomic stability and protect cells from harmful stress responses (Bonnet et al., 2017). Recent studies have led to the identification of yet another novel function of introns. Some excised introns provide protection against environmental stress (Morgan et al., 2019; Parenteau et al., 2019). Introns are also the source of snoRNA, microRNA, and lncRNA (Carmel and Chorev, 2012). These non-coding RNA species regulate gene expression at the level of transcription and RNA stability. Outside of acting as a non-coding regulatory RNA molecule, introns possess the unique function of acting as mutational buffers that can protect coding regions from incurring deleterious mutations (Jo and Choi, 2015). A harmful mutation in the coding region may affect the function of the protein. In contrast, a mutation in introns, which are non-coding regions, has minimal chances of affecting the function of the protein. In human genes, introns make up the bulk of a gene and therefore are able to absorb detrimental mutations without affecting the protein function. Although introns have been implicated in a variety of functions in eukaryotes, not all introns are associated with every function described above.

Of all the known functions of introns, one of the best known and evolutionarily conserved, is their ability to regulate the expression of genes that harbor them. Introns affect gene expression at multiple levels. They have been implicated in altering nucleosome positioning, transcription, RNA stability, nucleo-cytoplasmic export of mRNA, and translation efficiency (reviewed in Le Hir et al., 2003; Rose, 2008; Gallegos and Rose, 2015; Laxa, 2017; Shaul, 2017). The details regarding posttranscriptional regulation of gene expression by an intron have been covered in many recently published reviews (Gallegos and Rose, 2015; Laxa, 2017; Shaul, 2017). Here, we highlight the function of introns in regulating gene expression at the level of transcription. The transcription-enhancing potential of introns was first observed in cultured maize cells and transgenic mice (Callis et al., 1987; Brinster et al., 1988). Furthermore, cDNA of a number of human genes are not transcribed to the wild type level unless a promoter-proximal intron is included (Palmiter et al., 1991; Charron et al., 2007). Soon thereafter it was realized that introns play a general role in activating transcription in a variety of eukaryotes. Intron-mediated regulation of transcription has been observed in simple eukaryotes like yeast and Chlamydomonas as well as in higher eukaryotes like flies, worms, plants, and mammals including humans (Callis et al., 1987; Brinster et al., 1988; Bieberstein et al., 2012; Moabbi et al., 2012; Agarwal and Ansari, 2016; Baier et al., 2020). This enhancement property could be a very crucial function of introns in eukaryotes as a number of genes are dependent on introns for their normal transcription (Rose, 2019). In general, intron-containing genes in eukaryotes exhibit higher expression than their non-intronic counterparts (Ares et al., 1999; Juneau et al., 2006; Bieberstein et al., 2012; Gallegos and Rose, 2015; Ding and Elowitz, 2019; Baier et al., 2020). An intron may enhance transcription by a meager 2–3-fold, or it may augment mRNA output in the range of 10–100-fold or higher depending on the gene. Not all introns, however, can stimulate transcription. Some naturally occurring genes do not contain introns but are expressed efficiently, while some transgenes fail to express even in the presence of an intron (Pasleau et al., 1987; Malim et al., 1988; Rose, 2008). Introns are dispensable for enhancing transcription from a strong promoter (Huang and Liang, 1993). The transcription activation potential of introns though, is crucial for high expression of genes with a weak promoter. Some of the highly expressed genes like H2A and hepatitis B virus genes have *cis-*acting elements that appear to function like introns (Huang and Liang, 1993; Huang and Yen, 1995; Liu and Mertz, 1995). Despite some introns lacking the transcription enhancement potential, introns in general are emerging as an important component of the transcription regulatory machinery in eukaryotes (Rose, 2019).

Intron-mediated transcriptional regulation can be broadly divided into two categories: (1) splicing-independent, and (2) splicing-dependent regulation. Splicing-independent regulation is due to the presence of an enhancer or a promoter element within the intron (Kaneda et al., 1992; Bianchi et al., 2009; Beaulieu et al., 2011). Such introns can influence transcription even if their splicing function is compromised. In contrast, the splicing-dependent regulation requires a functional, splicing-competent intron within the transcribed region of the gene. Such introns cannot affect transcription if their splicing is inhibited by a mutation in the conserved sequences at the 5′ splice site, 3′ splice site and branchpoint, or if they are inserted in an anti-sense orientation (Furger et al., 2002; Moabbi et al., 2012; Agarwal and Ansari, 2016). This direct effect of a splicing-competent intron on transcription of a gene represents splicing-dependent regulation. It is often referred to as “intron-mediated enhancement” (IME). The focus of this review is to highlight key findings related to the possible mechanism of this enhancement with an emphasis on data indicating that genome architecture plays a critical role in the process.

## Intron-Mediated Enhancement Effect May Target Initiation, Elongation or Termination Steps

**FIGURE 2:**
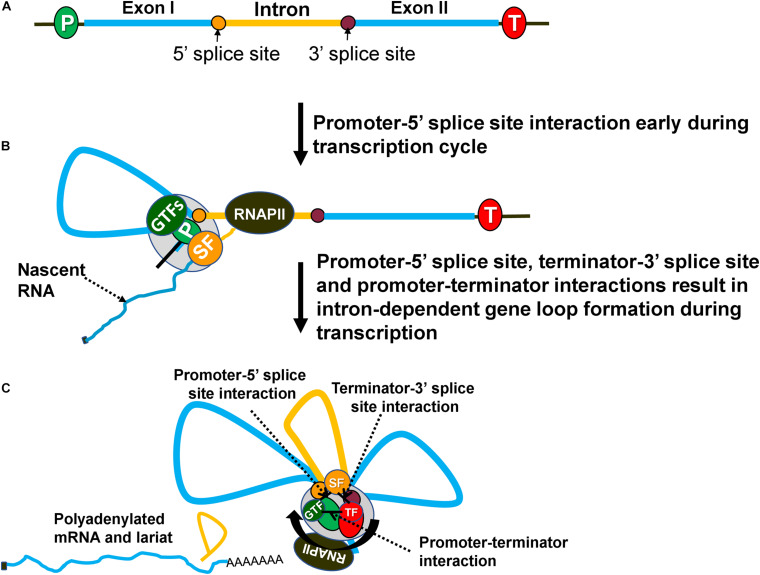
Intron-dependent gene looping enhances transcription by facilitating initiation, reinitiation and promoter directionality. **(A)** A gene with an intron (yellow) and exons (blue). **(B)** During transcription, an elongating RNAPII transcribes the gene and recruits splicing factors (SF) to splice out the intervening intronic sequence. Splicing components (SF) at the 5′ splice site interact with the general transcription factors (GTFs) at the promoter forming a loop between the promoter and 5′ splice site. **(C)** Once RNAPII has transcribed the intron, splicing components at the 3′ splice site associate with the termination factors near the 3′ end of the gene forming a loop between the 3′ splice site and terminator. Finally, the gene forms an overall three-looped conformation where the promoter and terminator physically interact with one another to assist in reinitiation. The three unique interactions that take place are between the promoter-terminator, promoter-5′ splice site and terminator-3′ splice site. GTFs, general transcription factors; SFs, splicing factors; TF, termination factors.

To enhance transcription, the intron must be located within the transcribed region of the gene and should be spliced (Figures 1A, 2A). The activation is maximum when an intron is present close to the promoter, and gradually decreases with increasing distance from the promoter (Callis et al., 1987; Rose, 2004; Bieberstein et al., 2012; Gallegos and Rose, 2017). There are, however, reports that in some genes, the activation potential of the intron is partially restored with increasing proximity to the terminator (Callis et al., 1987; Kim et al., 2011). Thus, the position of an intron within a gene is an important determinant of its transcription activation potential.

Although introns can impact any step of the transcription cycle to achieve higher mRNA output, the initiation step is the most frequent target. The transcription regulating ability of an intron is dependent on the cotranscriptional nature of splicing (Figure 1B). The carboxy-terminal domain (CTD) of RNA Polymerase II (RNAPII) is used as a recruitment and docking site for several splicing factors that help in coupling of transcription with splicing (Millhouse and Manley, 2005; Nojima et al., 2018). During cotranscriptional splicing, the spliceosome assembled on the elongating mRNA facilitates initiation/reinitiation by stabilizing assembly of the preinitiation complex (PIC) on the promoter (Figure 2B; Tian, 2001; Kwek et al., 2002; Das et al., 2007; Damgaard et al., 2008; Jobert et al., 2009). The 5′ splice site of nascent transcripts plays a crucial role in these processes. In fact, a 5′ splice site alone, without being a part of an intron, can enhance transcription to some extent (Damgaard et al., 2008). The 5′ splice site enhances recruitment of TFIID, TFIIB, and TFIIH on the promoter (Das et al., 2007; Damgaard et al., 2008). Splicing factors like U1-snRNP and HNRNPU, which bind to the 5′ splice site, facilitate the recruitment of these general transcription factors (Tian, 2001; Das et al., 2007). U1-snRNP physically interacts with RNAPII during cotranscriptional splicing (Nojima et al., 2018). U1-snRNP also contacts the cyclin H subunit of TFIIH (Kwek et al., 2002). U1-snRNP may facilitate initiation and reinitiation of transcription by enabling recruitment of TFIIH, and probably TFIID and TFIIB as well (Kwek et al., 2002; Damgaard et al., 2008). HNRNPU similarly promotes initiation by enabling the recruitment of TFIIF to the pre-initiation complex (PIC) (Fiszbein et al., 2019). Furthermore, the promoter-proximal introns also affect histone H3-K9 acetylation and H3-K4 trimethylation near the 5′ end of genes (Figure 1B; Bieberstein et al., 2012). These two chromatin modifications facilitate the recruitment of the PIC on the promoter leading to the activation of genes, both in yeast and higher eukaryotes. Thus, an intron may affect initiation directly by facilitating the recruitment of general transcription factors on the promoter or indirectly by affecting the chromatin structure in the promoter region.

Introns have also been found to stimulate transcription at the elongation as well as termination steps of transcription. Many independent studies have found U1-snRNP, SKIP and SC35, which are splicing factors, stimulating elongation by interacting with various transcription elongation factors (Fong and Zhou, 2001; Brès et al., 2005; Lin et al., 2008). Introns also influence elongation indirectly by affecting chromatin structure. The H3-K36 trimethylation mark, which is specifically associated with elongation of transcription, is enriched in intron-containing genes compared to the intron-less genes (Figure 1B; De Almeida et al., 2011). There are also a few reports of terminator-proximal introns influencing transcription by affecting termination of transcription either directly by helping in the recruitment of termination factors or indirectly by affecting the chromatin structure near the 3′ end of genes (Lutz et al., 1996; McCracken et al., 2002). The 3′ splice site of a terminator-proximal intron has been shown to enhance utilization of a downstream poly(A) site by facilitating recruitment of the F 3′ end processing complex and poly(A) polymerase in mammalian cell lines (Figure 1B; Lutz et al., 1996; McCracken et al., 2002). The 3′ splice site also has an adverse effect on H3-K36 trimethylation, which is a transcription elongation mark that needs to be removed to facilitate termination of transcription (Kim et al., 2011). Although introns may target the elongation and termination steps to enhance transcription, the initiation step is emerging as the most frequent target.

## Intron-Dependent Looped Gene Architecture Facilitates Enhancement of Transcription

The classical view of transcriptional regulation by *cis*-acting regulatory sequences and *trans*-acting protein factors has undergone radical changes due to research carried out during the last few decades (Papantonis and Cook, 2010; Ansari, 2019; Al-Husini et al., 2020). Genome topology or chromatin conformations formed by enhancer-promoter interactions and promoter-terminator interactions have been found to play a crucial role in regulation of transcription (Misteli, 2007). The physical interaction of the promoter and terminator regions of a gene during transcription results in the formation of a looped gene architecture (Ansari and Hampsey, 2005). Such gene loops are formed in an activator-dependent manner and have been observed in yeast as well as in higher eukaryotes (reviewed in Al-Husini et al., 2020). Activator-dependent gene looping has been shown to enhance transcription by facilitating direct transfer of polymerase from the terminator to the promoter for reinitiation, and by enhancing promoter directionality (Tan-Wong et al., 2012; Al Husini et al., 2013).

In budding yeast, the presence of an intron in a gene also results in a looped gene architecture (Figure 2C; Moabbi et al., 2012; Tan-Wong et al., 2012; Agarwal and Ansari, 2016). Intron-dependent gene looping, however, is mechanistically different from the activator-dependent looped conformation. The “Chromosome Conformation Capture” (3C) approach revealed that an intron-dependent loop is characterized by additional contacts of the promoter with the 5′ splice site and of the terminator with 3′ splice site (Figures 2B,C; Moabbi et al., 2012). The promoter-5′ splice site loop may not be observed in all genes as the first intron often is located very close to the promoter and can make direct contact with the promoter without loop formation. How the presence of an intron facilitates gene loop formation, however, is not yet clear. It has been proposed that the interaction of a 5′ splice site with the promoter and of a 3′ splice site with terminator bring the two ends of a gene in close physical proximity and facilitate the promoter-terminator contact (Al-Husini et al., 2020). Only a splicing-competent intron facilitates gene loop formation (Moabbi et al., 2012; Agarwal and Ansari, 2016). Mutation of either the 5′ or the 3′ splice sites abolishes intron-facilitated looped gene structure. Since the mutation of splice sites also adversely affects IME of transcription, it was proposed that intron-mediated gene looping may also enhance transcription in a manner similar to activator-dependent enhancement. Examination of the IME effect in the looping-defective mutants of yeast revealed that although splicing was normal, there was no enhancement of transcription (Moabbi et al., 2012; Agarwal and Ansari, 2016). The IME effect therefore is not due to splicing *per se* but due to the formation of a splicing-dependent looped gene architecture in budding yeast.

Evidence suggests that the three contact points in an intron-dependent looped gene structure; promoter-terminator contact, promoter-5′ splice site contact and terminator-3′ splice site (Figure 2C), are established by a protein-protein interaction of factors occupying promoter, terminator and intronic sites. A combination of ChIP and 3C approaches identified the crucial role of the general transcription factor TFIIB and CF1A termination complex in the promoter-terminator interaction (Medler et al., 2011). Similarly, the interaction of another general transcription factor TFIIH with U1-snRNP could lead to promoter-5′ splice site contact (Kwek et al., 2002). The interactions contributing to terminator-3′ splice site contact are yet to be established. Splicing factors do not contact DNA directly, but proximity of splice sites on RNA with the corresponding DNA region during cotranscriptional splicing results in splicing factors getting crosslinked to the splice sites on DNA as well (Kotovic et al., 2003; Lin et al., 2008; Oesterreich et al., 2016; Minocha et al., 2018; Nojima et al., 2018). This evidence suggests that the intron-mediated gene loop formation is due to an interaction of the initiation and termination factors occupying distal ends of a gene with splicing factors bound to the intronic regions.

A critical issue is how intron-dependent looped gene architecture brings about enhancement of transcription. All three of the physical interactions in an intron-facilitated gene loop have the potential to enhance transcription. The promoter-5′ splice site interaction can facilitate initiation/reinitiation by stabilizing the assembly of the PIC (Figure 2B). U1-snRNP, which binds the 5′ splice site and exhibits an interaction with TFIIH, may play a crucial role in this regard (Kwek et al., 2002). U1-snRNP may directly help in the recruitment of TFIIH, and possibly TFIIB and TFIID on the promoter if the 5′ splice site is located in close proximity to the promoter (Damgaard et al., 2008). The 5′ splice sites, however, may be located several hundred nucleotides away from the promoter. In such a scenario, a loop formed by promoter-5′ splice site interaction may play a critical role in assembly or stabilization of the PIC on the promoter (Figure 2B). The net result will be enhanced initiation or reinitiation of transcription. This may explain why a functional 5′ splice site alone could bring about an increase in transcription of HIV-1 and β-globin genes (Damgaard et al., 2008). The enhancement of transcription elicited by a 5′ splice site alone, however, was much lower (75% less) compared to that brought about by a full-length intron. The interaction of the 5′ splice site with the promoter is therefore not sufficient to achieve enhancement of transcription fully. Contacts of the promoter with the terminator and of the 3′ splice site with the terminator may contribute significantly to the IME effect (Figure 2C). Thus, all three interactions in an intron-dependent gene loop may play a role in the enhancement of transcription.

The promoter-terminator interaction is especially crucial as it can enhance transcription by facilitating reinitiation and by conferring promoter directionality (Tan-Wong et al., 2012; Al Husini et al., 2013). It is conceivable that proximity of promoter and terminator in the intron-dependent gene loop may similarly contribute to the enhancement of transcription by a similar mechanism (Figure 2C). Juxtaposition of the terminator and promoter facilitates release of polymerase from the terminator region near the promoter. The polymerase is then recycled back to the juxtaposed promoter for reinitiation of transcription (Al Husini et al., 2013). Such a coupling of termination to reinitiation, with a concomitant increase in the transcriptional activity, has been observed for RNAPIII, RNAPI, mitochondrial polymerase and archaeal polymerase (Dieci and Sentenac, 1996; Jansa et al., 2001; Martin et al., 2005; Spitalny and Thomm, 2008). The promoter-terminator interaction is emerging as a critical player in regulation of transcription in eukaryotes.

In budding yeast, juxtaposition of the promoter and terminator also confers promoter directionality, which is the enhancement of promoter-initiated transcription of mRNA while keeping the upstream antisense transcription in check (Tan-Wong et al., 2012; Al Husini et al., 2013). The presence of an intron also enhances promoter directionality of yeast genes (Agarwal and Ansari, 2016). In the absence of a splicing-competent intron, mRNA synthesis exhibits a decline, while uaRNA (upstream antisense RNA) transcription increases. In a looping-defective strain, despite the presence of a splicing-competent intron and normal splicing, promoter directionality is adversely affected. The proximity of the promoter and terminator in the intron-mediated gene loop allows the termination factors bound to the 3′ end to contact the 5′ end of a gene and bring about termination of uaRNA transcription (Agarwal and Ansari, 2016). The net result is higher transcription of the gene by enhancing promoter directionality.

Although, the terminator-3′ splice site interaction can promote cotranscriptional recruitment of termination factors, leading to efficient termination of transcription, its role in IME effect needs further exploration. It is, however, clear from the studies described above that intron-dependent looped gene architecture likely plays a crucial role in enhancement of transcription in budding yeast.

## Splicing-Dependent Gene Looping in Higher Eukaryotes

In budding yeast, merely 4% of genes contain introns, but these few genes produce more than 25% of total cellular mRNA (Ares et al., 1999). On average, yeast intron-containing genes produce 3.7 times more mRNA than their non-intronic counterparts (Juneau et al., 2006). In higher eukaryotes, a far higher proportion of genes harbor introns, and splicing-dependent activation of transcription is prevalent in higher eukaryotes as well (reviewed in Rose, 2008; Laxa, 2017; Shaul, 2017; Rose, 2019). This raises the question if splicing-associated changes in gene architecture also occur in higher eukaryotes, and if they contribute to enhancement of transcription.

Chromatin interaction analyses have revealed the presence of physical interactions of promoters with their gene body in mammalian systems. 3C analysis of the human BRCA1 gene revealed an interaction of the promoter and terminator regions of the gene with the intronic regions in a transcription-dependent manner (Tan-Wong et al., 2008). Intragenic gene loops formed by the interaction of exons with cognate promoters have been identified in human cell lines on a genomewide scale using the ChIA-PET (Chromatin Interaction Analysis with Paired-End Tag) approach (Mercer et al., 2013). Hi-C analysis corroborated the presence of intragenic chromatin loops formed by the interaction of promoters with exons in the human genome (Ruiz-Velasco et al., 2017). This genomewide study also demonstrated intragenic gene loops between exons and the 3′ ends of genes. This is reminiscent of the intron-terminator interaction observed in budding yeast and the human BRCA1 gene (Tan-Wong et al., 2008; Moabbi et al., 2012). ChIA-PET identified CTCF (CCCTC-binding factor) as the protein that facilitates the interaction of promoter with exons (Mercer et al., 2013). It was further shown that the CTCF-mediated promoter-exon loops are prevalent in genes coding for proteins involved in cell signaling and response to stimuli. The promoter-exon loop formation is accompanied by trimethylation of histone H3-K4, acetylation of histone H3-K27 and trimethylation of histone H3-K36 in the coding region of the gene (Ruiz-Velasco et al., 2017). These three histone modifications are associated with transcriptionally active chromatin (Bannister and Kouzarides, 2011). Modification of histone H3-K4 and H3K9 by methylation and acetylation have been implicated in enhancement of transcription by the promoter-proximal intron in mammalian cells thereby suggesting that the promoter-exon interaction may enhance transcription by affecting chromatin structure (Bieberstein et al., 2012). The promoter-exon interaction may be the mammalian structural equivalent of yeast intron-mediated gene loops.

The promoter-exon interaction may be responsible for recently reported internal exon-mediated activation of transcription in mammalian cells (Fiszbein et al., 2019). Internal exons are small exons generally less than 300 nucleotides in length and are flanked by at least one exon on the 5′ side and one exon on the 3′ side. They are responsible for transcriptional regulation of thousands of mammalian genes. The phenomenon is called EMAT (exon-mediated activation of transcription). Activation of transcription by internal exons is not dependent on the sequence of the exon, but on the splice sites flanking the intron. Mutation of either the 5′ or 3′ splice site completely abrogated transcription activation potential of an internal exon (Fiszbein et al., 2019). Furthermore, exon-mediated activation involved recruitment of the general transcription factor TFIIF on the promoter by the splicing factor HNRNPU (heterogeneous nuclear ribonucleoprotein U), which is known to interact with TFIIF through its N-terminal domain (Kim and Nikodem, 1999). In addition, the exon-promoter interaction is accompanied by trimethylation and acetylation of histone H3, which may also result in enhanced transcription by affecting both transcription initiation and elongation (Bieberstein et al., 2012; Ruiz-Velasco et al., 2017).

The exon-mediated transcriptional regulation in mammalian cells exhibits striking similarities to the intron-mediated regulation in budding yeast: (1) internal exon or intron must be located within one kbp of the promoter to activate transcription; (2) transcription activation by both occurs in a splicing-dependent manner; and (3) transcriptional regulation in both cases involves splicing-dependent recruitment of general transcription factors on the promoter. The possibility of exon-mediated transcriptional activation through intragenic gene loops formed by the interaction of a promoter with the gene body therefore cannot be ruled out. Both intron-mediated and exon-mediated enhancement of transcription are in fact splicing-mediated regulations. Gene architecture playing a general role in splicing-mediated regulation of transcription is an attractive possibility. The studies from yeast strongly suggest the involvement of gene looping in the splicing-mediated regulation, but evidence from higher eukaryotes are still preliminary and needs further investigation. Nevertheless, it is clear that introns are not merely junk coding sequence but are an important regulator of cellular functions.

## Author Contributions

AA conceptualized. KD and NA prepared the original draft. KD made figures and helped with editing. AA and LP edited the manuscript. All authors contributed to the article and approved the submitted version.

## Conflict of Interest

The authors declare that the research was conducted in the absence of any commercial or financial relationships that could be construed as a potential conflict of interest.
